# *C. elegans *feeding defective mutants have shorter body lengths and increased autophagy

**DOI:** 10.1186/1471-213X-6-39

**Published:** 2006-08-03

**Authors:** Catarina Mörck, Marc Pilon

**Affiliations:** 1 Dept. Cell and Molecular Biology, Göteborg University, Box 462, SE-405 30 Göteborg, Sweden

## Abstract

**Background:**

Mutations that cause feeding defects in the nematode *C. elegans *are known to increase life span. Here we show that feeding defective mutants also have a second general trait in common, namely that they are small.

**Results:**

Our measurements of the body lengths of a variety of feeding defective mutants, or of a variety of double mutants affecting other pathways that regulate body length in *C. elegans*, i.e. the DBL-1/TGFβ, TAX-6/calcineurin and the SMA-1/β_H_-spectrin pathways, indicate that food uptake acts as a separate pathway regulating body length. In early stages, before eating begins, feeding defective worms have no defect in body length or, in some cases, have only slightly smaller body length compared to wild-type. A significant difference in body length is first noticeable at later larval stages, a difference that probably correlates with increasing starvation. We also show that autophagy is induced and that the quantity of fat is decreased in starved worms.

**Conclusion:**

Our results indicate that the long-term starvation seen in feeding-defective *C. elegans *mutants activates autophagy, and leads to depletion of fat deposits, small cell size and small body size.

## Background

**Figure 1:**
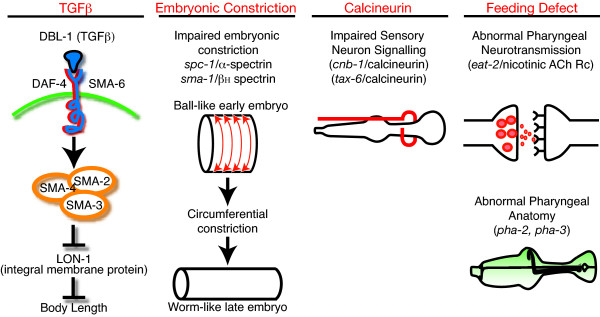
**Genetics of body length in *C. elegans***. The four major pathways that regulate body length in *C. elegans *are illustrated in general terms and described in more details within the introduction.

It is obvious that body size in the nematode *C. elegans *is genetically regulated: many mutations in *C. elegans *result in abnormal body sizes. Examples include mutations that affect cuticle collagen and cause Dumpy (Dpy) phenotypes [1]. There is a number of genes that seem to regulate body size in a more indirect manner resulting in Small (Sma) or Long (Lon) worms. According to the literature, there appears to be at least three different genetic pathways that determine *C. elegans *body length (see Fig. 1 and Table 1). These are: 1) A TGF-β pathway, involving the *dbl-1, sma-2, sma-3, sma-4*, *kin-29*, *lon-1 *and additional genes [2-5]; 2) A spectrin pathway involving the *sma-1 *[6], *spc-1 *[7] and *unc-70 *[8] genes and 3) A calcineurin pathway involving the calcineurin homologs *tax-6 *[9] and *cnb-1*, both expressed in many sensory neurons and most muscle cells [10], where expression of *tax-6 *specifically in neurons rescues the small body phenotype in the *tax-6 *mutant [9]. Some mutations that cause small worms have not yet been firmly assigned to these pathways. For example, *rnt-1*, which encodes a homolog to the mammalian RUNX transcription factors, has been assigned to the TGF-β pathway but this conclusion rests on genetic interaction experiments still compatible with other interpretations [11,12]. Also, the *sma-5 *mutant exhibits a small, thin and slow growth phenotype, and was recently found to encode a homolog to the MAP kinase BMK1/ERK5. This gene is expressed in the intestine, hypodermis, excretory cell and the pharynx, and the body length defects are additive to the DBL-1/TGF-β pathway [13]. It will therefore be interesting in the future to find out if *sma-5 *is part of another yet undiscovered pathway regulating body size besides the ones described here (Fig 1).

**Table 1:** Genes involved in body length determination that were part of the present study.

**Genotype**	**Allele**	**Protein**	**Pathway**	**Ref.**
*dbl-1*	null	TGF-β growth factor	TGF-β	[44]
*eat-1*	unknown	α-actinin associated LIM protein	Feeding	[45]
*eat-2*	null	nicotinic acetylcholine receptor subunit	Feeding	[38]
*eat-3*	unknown	dynamin-like GTP binding protein	Feeding	[45]
*eat-10*	unknown	unknown	Feeding	[45]
*egl-4*	g.o.f*	cGMP dependent protein kinase	TGF-β	[45, 46]
*kin-29*	null	serine/threonine kinase	TGF-β	[47]
*lon-1*	e185* wk50*	PR (pathogenesis related)-protein	TGF-β	[48]
*pha-2*	l.o.f*	homeodomain transcription factor	Feeding	[39]
*pha-3*	unknown	unknown	Feeding	[45]
*rnt-1*	deletion*	RUNX transcription factor	not determined	[11]
*sma-1*	null	βH-spectrin	Spectrin	[6]
*sma-2*	missense*	TGF-β receptor signalling protein	TGF-β	[49]
*sma-3*	missense*	TGF-β receptor signalling protein	TGF-β	[49]
*tax-6*	missense**	serine/threonine protein phosphatase	Calcineurin	[9]

g.o.f* (gain of function alleles): *egl-4(ad450) *gain of function mutation, e185*: *lon-1(e185) *is considered to be a null allele. wk50*: *lon-1(wk50) *mutation cause a premature stop producing a 193 amino acid protein. l.o.f* (loss of function alleles): *pha-2(ad472*) is a pharynx specific loss of expression allele. Deletion*: in *rnt-1(ok351) *38 amino acids are deleted, included parts of the DNA binding domain, mRNA is still produced. Missense*: both *sma-2(e502) *and *sma-3(e491) *contains missense mutations and the alleles are considered to be strong loss of function. Missense**: *tax-6 (p675) *is a missense mutation allele, complete or nearly loss of function

While the genetic basis of body size in *C. elegans *is beginning to be well understood, it is perhaps less well appreciated that the life experience of this organism can also influence body size. It is well known that caloric restriction can increase life span in *C. elegans *or induce formation of the growth arrested *dauer *larva [14,15]. Lakowski *et al *[14] showed that mutations in many of the *eat *mutant genes lengthens the life span: *eat-1(ad427) *lives 33% longer, *eat-2 (ad465) *29%, *eat-3 (ad426) *11% and *eat-10 (ad606) *8% longer. They also showed that there is a correlation between longer life span and the severity of the feeding defect: the *eat-1 *allele with the slowest pumping rates *(ad427) *lives 33% longer while the weakest allele *(e2343) *lives 11% longer.

However, very few reports have emphasized the fact that reduced food uptake also correlates with reduced body length. In one report, a feeding defective mutant (*eat-2*) was shown to have a smaller body volume than wild-type worms, but no actual length measurements were reported [16]. In another study, a deletion in the intestinal peptide transporter gene *pep-2*, which effectively reduces the delivery of amino acids for growth and development from the gut lumen into the intestinal cells, was found to have a reduced body length, [17]. Given the great interest in correlating the effect of food deprivation on longevity [14,18,19], as well as the strong inverse relationship between height and longevity in humans [20], we decided to determine whether body size is also inversely correlated with food uptake in the organism *C. elegans*, a popular model to study longevity.

Here we show that feeding defective mutants are short, independently of the nature of the genetic defect that impairs the feeding behavior. This suggests that food uptake acts as a separate pathway regulating body length. We also show that fat stores are depleted but that autophagy is upregulated in feeding defective mutants. Autophagy is a catabolic process through which proteins and organelles are degraded and the amino acids are reused by the cells. Constituents to be degraded are engulfed by double-membrane cytoplasmic vesicles forming an autophagosome that further fuses with lysosomes for degradation (for review see [21,22]). In the fruit fly *Drosophila Melanogaster *it has been reported that starvation induces autophagy in the larval fat body [23]). In *C. elegans*, autophagy was previously known to occur before entry into the dauer larval stage and the process is thought to be involved in the remodelling of various tissues of the dauer larva [24].

## Results

### Feeding defective mutants are short and thin

**Figure 2:**
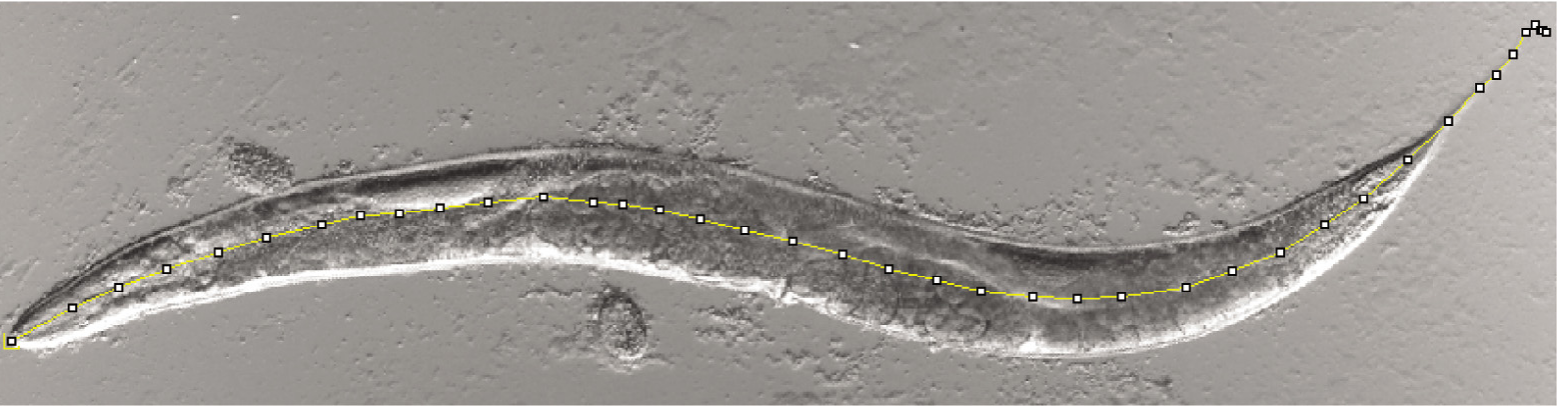
**Example of body length measurement using the ImageJ software**. The black squares indicate the location of mouse clicks while the connecting yellow line is generated by the ImageJ software. Total length is the sum of the length of the yellow lines, and is calibrated within the software using an image of a scale bar as reference.

**Figure 3:**
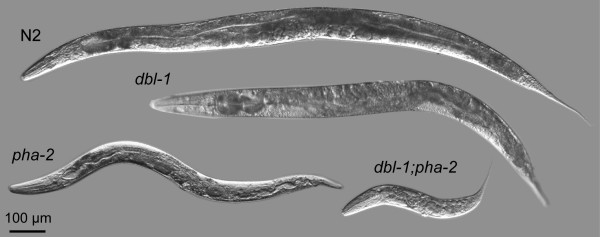
**Photographs of N2, *dbl-1*, *pha-2 *and *dbl-1;pha-2 *representative animals**. Note that while the *pha-2 *and *dbl-1 *animals are of approximately the same length, the *pha-2 *is comparatively thinner. Note also that the double mutant is remarkably short and with a body width similar to that of the *pha-2 *single mutant. All worms were developmentally age-matched to 48 hours post L4 stage.

We measured the length of photographed age-matched *C. elegans *adults of a variety of genotypes and found that all the feeding defective mutants that we studied were significantly shorter than wild-type animals (Fig. 2, 3 and Table 2). In particular, we found that mutants with abnormal pharyngeal anatomies (*pha-2, pha-3*), or normal pharynxes with reduced pumping rates (*eat-1, eat-2, eat-3*) or with inefficient pharyngeal pumping (e.g. slippery pharynx that inefficiently traps bacteria; *eat-10*) were all short.

**Table 2:** Body length and width measurements of single and double mutants. The body lengths and widths were measured 48 hrs after L4 larval stage, 20–26 worms were studied per genotype. nd – not determined.

**Genotype **(in alphabetic order after N2)	**Body length μm **(SEM)	**% body length**	**Body width μm **(SEM)	**% body width**
N2	1424 (± 2,4)	100	82(± 0,1)	100
*dbl-1(nk3)*	985 (± 2,2)	69	79(± 0,3)	96
*dbl-1;eat-1*	594 (± 2,1)	42	nd	-
*dbl-1;eat-3*	1002 (± 4,2)	70	nd	-
*dbl-1;lon-1(wk50)*	1211 (± 4,3)	85	nd	-
*dbl-1;pha-2*	421 (± 1,9)	30	44(± 0,2)	54
*dbl-1;sma-1*	larval lethal	-	nd	-
*eat-1(ad427)*	757 (± 1,5)	53	48(± 0,1)	59
*eat-2(ad465)*	946 (± 2,9)	66	63(± 0,2)	77
*eat-3(ad426)*	1042 (± 4,3)	73	64(± 0,3)	78
*eat-3;kin-29*	945 (± 3,6)	66	nd	-
*eat-3;lon-1(wk50)*	1180 (± 2,8)	83	nd	-
*eat-3;pha-2*	larval lethal	-	nd	-
*eat-3;rnt-1*	larval lethal	-	nd	-
*eat-3;sma-1*	723 (± 3,3)	51	nd	-
*eat-3;sma-2*	571 (± 2,7)	40	nd	-
*eat-3;sma-3*	657 (± 5,2)	46	nd	-
*eat-3;tax-6*	785 (± 2,8)	55	nd	-
*eat-10(ad606)*	1041 (± 3,2)	73	58(± 0,2)	71
*egl-4(ad450)*	947 (± 2,3)	67	61(± 0,2)	74
*kin-29(oy38)*	885 (± 3,9)	62	nd	-
*kin-29;pha-2*	672 (± 3,6)	47	nd	-
*lon-1(e185)*	1450 (± 3,2)	102	nd	-
*lon-1;pha-2*	966 (± 7,4)	68	nd	-
*lon-1(wk50)*	1300 (± 3,5)	91	nd	-
*lon-1(wk50);pha-2*	1042 (± 5,9)	73	nd	-
*pha-2(ad472)*	902 (± 4,0)	63	61(± 0,2)	74
*pha-2;rnt-1*	larval lethal	-	nd	-
*pha-2;sma-1*	861 (± 3,4)	60	nd	-
*pha-2;sma-2*	larval lethal	-	nd	-
*pha-2;sma-3*	larval lethal	-	nd	-
*pha-2;tax-6*	877 (± 4,3)	62	nd	-
*pha-3(ad607)*	707 (± 2,2)	50	47(± 0,2)	57
*rnt-1(ok351)*	1250 (± 2,9)	88	83(± 0,2)	101
*sma-1(ru18)*	1044 (± 2,7)	73	81(± 0,2)	99
*sma-1;tax-6*	523 (± 2,8)	37	nd	-
*sma-2(e502)*	692 (± 2,2)	49	59(± 0,2)	72
*sma-3(e491)*	752 (± 2,7)	53	63(± 0,2)	77
*tax-6(p675)*	858 (± 2,5)	60	63(± 0,2)	77

As control for our general methodology we evaluated the length of wild-type N2 worms and found it to be the same as published values. Also, we scored a variety of other small mutants that fall within the TGF-β pathway (*dbl-1*, *egl-4*, *sma-2*, *sma-3 *and *kin-29*), the spectrin pathway (*sma-1*) or the calcineurin pathway (*tax-6*), as well as the mutant *rnt-1*: as expected, all of these mutants were short (Table 2). Finally, the *lon-1 *(*e185) *null allele caused worms to be slightly longer than wild-type, as expected (Table 2).

We also measured the body width of the feeding-defective mutant and of other single mutants with a small phenotype (*dbl-1, egl-4, kin-29, rnt-1, sma-1, sma-2, sma-3 *and *tax-6*) and one double mutant (*dbl-1;pha-2*) (Fig. 3 and Table 2). All feeding defective mutants were significantly thinner than wild-type, as were *sma-2, sma-3 *and *tax-6*. *dbl-1 *worms were only slightly thinner and *sma-1 *and *rnt-1 *worms exhibited wild-type widths. The thinnest worms were the *dbl-1*;*pha-2 *double mutants, which indicates an additive effect between these two mutations.

### Food uptake efficiency acts as a separate pathway regulating body length

If two separate mutations cause reduced body length via separate pathways, one would expect that an individual worm homozygous for both mutations should be much smaller than either single mutant alone: the two mutations would show an additive effect. It is even possible that the additive effects of two such mutations might result in lethality, if their combined effect is too severe. We generated several double mutants and found that the results generally agree with the hypothesis that food uptake efficiency is a separate pathway regulating body length that shows an additive effect with the other established pathways. In particular, mutations in the DBL-1/TGF-β pathway showed an additive effect on body length (or even caused larval lethality) when combined with the pharyngeal abnormal mutation *pha-2 *(e.g. *dbl-1;pha-2, kin-29;pha-2, pha-2;sma-2, pha-2;sma-3*) and the pharyngeal pumping mutants *eat-1 *(e.g. *eat-1;dbl-1*) and *eat-3 *(e.g. *eat-3;sma-2, eat-3;sma-3*) (Table 2). Similarly, combining *pha-*2 or *eat-3 *with the β_H _spectrin *sma-1 *mutation or the *rnt-1 *mutation resulted in shorter worms or larval lethality (Table 2). Also consistent with the additive effects of combining mutations from distinct length-regulating pathways were the consequences of creating *dbl-1;sma-1 *and *sma-1;tax-6 *double mutants, which were both smaller than the single mutants involved (Table 2). Finally, we found that combining two separate mutations that impair food uptake via different mechanisms, and thus potentially completely prevent feeding, can cause larval lethality, as with the *eat-3;pha-2 *double mutant.

If one mutation causes an increase in body length by interfering with one pathway while a second mutation causes a reduced body length by interfering with a separate pathway, one would expect the double mutant to show an additive effect and thus to be of intermediate length. Morita *et al *previously reported that double mutants of *lon-1(e185) *and *dbl-1(nk3) *had intermediate body length, as if these genes belonged to separate pathways [24]. In contrast, the weaker *lon-1(wk50) *allele behaves as if *lon-1 *and *dbl-1 *were in the same pathway: the double mutant *lon-1(wk50) dbl-1(nk3) *results in worms longer than wild-type, indicating that *lon-1 *is epistatic to *dbl-1 *[25]. Given these premises, we used both *lon-1(e185) *and *lon-1(wk50) *to test whether feeding acts separately from the DBL-1/TGFβ pathway in regulating body length. This was indeed the case: the *eat-3;lon-1 (wk50)*, *lon-1(e185);pha-2 *and *lon-1 (wk50);pha-2 *double mutants had intermediate lengths compared to their constituent single mutants (Table 2). (Note that in our study, the *dbl-1;lon-1(wk50) *double mutant showed an additive effect of the mutations, suggesting that *lon-1 *is not completely epistatic to *dbl-1*.)

Three of the double mutants tested behaved in unexpected ways: the *dbl-1;eat-3*, *eat-3;kin-29 *and *pha-2;tax-6 *double mutants showed no additive effect between the individual two mutations involved (Table 2). This may possibly reflect overlaps in branching pathways between these mutants.

### The difference in body length is most prominent at later larval stages

**Figure 4:**
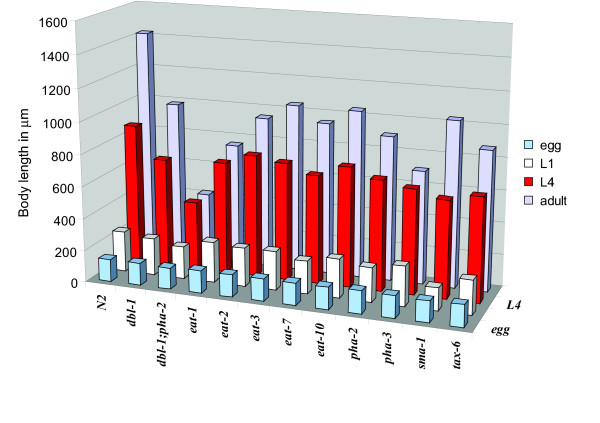
**Body length in eggs, larvae and adults of various genotypes**. Each bar shows the average of at least 25 measured individuals. See Table 3 for exact values and standard error of the mean.

Some of the mutants studied were dramatically smaller than wild-type worms, especially double mutants such as *dbl-1;pha-2 *that are only 30% of wild-type in length (Fig. 3). We studied the eggs of small mutants to determine if they are also reduced in size. Specifically, we measured the circumference of eggs containing embryos of the same developmental stage for the mutants *dbl-1*, *dbl-1;pha-2*, *eat-1*, *eat-3*, *eat-10*, *pha-2*, *pha-3*, *sma-1 *and *tax-6 *(Fig. 4 and Table 3). Only *dbl-1;pha-2 *and *sma-1 *showed a minor decrease in size.

**Table 3:** Study of eggs, L1 and L4 length. Eggs containing 3-fold stage embryos were measured. L1 larvae were measured within 1 hr after hatching and L4 larvae scored had an obvious white crescent surrounding the prospective vulva. At least 25 individuals were studied per genotype and experiment.

**Genotype **(in alphabetical order after N2)	**Egg circumference μm **(SEM)	**% circumference**	**L1 length μm **(SEM)	**% length**	**L4 length μm **(SEM)	**% length**
N2	138(± 0,2)	100	253(± 0,4)	100	878 (± 2,0)	100
*dbl-1(nk3)*	139(± 0,2)	101	230(± 0,7)	91	677 (± 2,6)	77
*dbl-1;pha-2*	132(± 0,2)	96	202(± 0,8)	80	423 (± 1,6)	48
*eat-1(ad427)*	140(± 0,2)	101	254(± 0,9)	100	696 (± 1,7)	79
*eat-2(ad465)*	139(± 0,2)	101	240(± 0,7)	95	762 (± 3,1)	87
*eat-3(ad426)*	138(± 0,3)	100	241(± 0,8)	95	734 (± 1,7)	84
*eat-10(ad606)*	139(± 0,2)	101	243(± 0,8)	96	747 (± 1,9)	85
*egl-4(ad450)*	138(± 0,2)	100	207(± 0,6)	82	676 (± 1,4)	77
*pha-2(ad472)*	141(± 0,2)	102	214(± 0,8)	85	688 (± 2,2)	78
*pha-3(ad607)*	138(± 0,2)	100	247(± 0,6)	98	654 (± 2,4)	75
*sma-1(ru18)*	132(± 0,2)	96	140(± 0,4)	55	607 (± 2,1)	69
*tax-6(p675)*	136(± 0,2)	99	213(± 0,7)	84	649 (± 1,4)	74

To investigate at which stage of postembryonic development the difference in body length between wild-type and the feeding defective mutants are first prominent we measured the body length at two larval stages, L1 and L4. In this study we also included some of the mutants from the other pathways (*dbl-1*, *sma-1 *and *tax-6*) and one double mutant (*dbl-1;pha-2*) (Fig. 4 and Table 3).

L1 larvae from all the feeding mutants, except *eat-1 *and *sma-1*, were only slightly smaller than wild-type larvae, and so were the *dbl-1 *and *tax-6 *mutants. In a previous report *sma-1(ru18) *was shown to exhibit a small phenotype already at the embryo stage due to elongation defects [6], an observation that we here confirmed: *sma-1 *worms are significantly smaller already at the L1 stage. The double mutant *dbl-1;pha-2 *is also shorter than wild-type but only a small additive effect is seen at this stage compared to the single mutants.

At the L4 stage all the feeding-defective and other small mutants are much smaller than wild-type, a difference that persists in adults (Fig. 4, Table 2 and 3). In conclusion, most of the short body length phenotype of the feeding-defective mutants develops once feeding has begun.

### Autophagy is induced in feeding defective mutants

Growth of cells and tissues during development is influenced not only by anabolic processes such as protein synthesis, but also by the rate and extent of catabolic, degradative processes. The turnover of most long-lived cellular proteins occurs through a process known as autophagy (reviewed in [26]). In this process, cytoplasm and organelles are non-selectively engulfed by a double membrane-bound vesicle, the autophagosome, which fuses with the lysosomal and/or endosomal compartment. The resulting degradation products can be a source of cellular nutrients, and indeed autophagy is strongly induced in response to nutrient deprivation in many cell types. Autophagy is also activated in *C. elegans *lavae that are entering the long-lived dauer state in response to adverse conditions [24].

**Figure 5:**
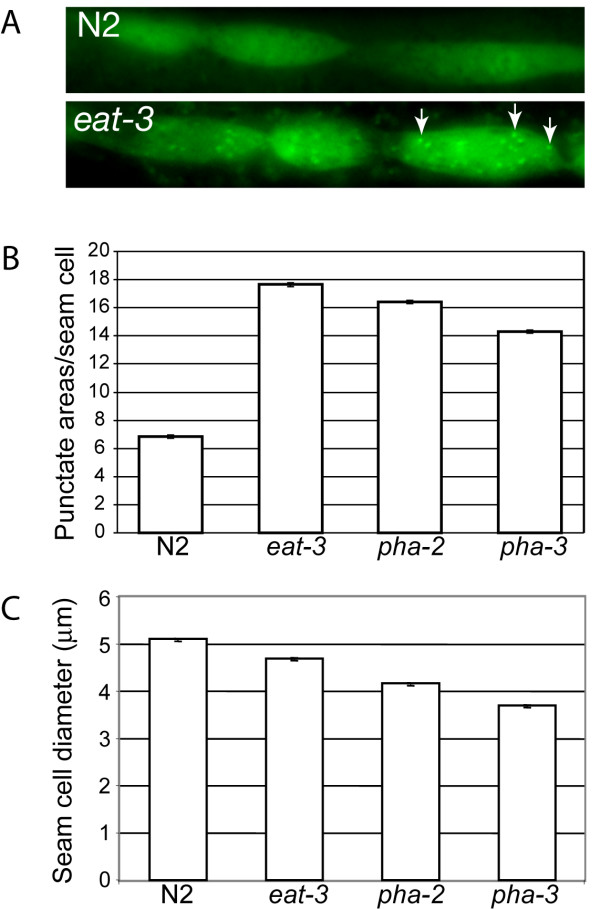
**Autophagy study in wild-type and the feeding defective mutants *eat-3*, *pha-2 *and *pha-3***. Worms transgenic for the autophagy marker *lgg-1::gfp *were studied at the L3 stage. (A) Expression of *lgg-1::gfp *in the seam cells is diffuse in wild-type with few punctate areas. In the feeding defective mutants represented here by *eat-3*, many GFP-positive punctate areas are found in the cytoplasm of the seam cells (some are indicated by arrows). (B) *lgg-1::gfp *positive punctate areas (preautophagosomal and autophagosomal structures) were counted in the hypodermal seam cells in at least 50 cells per strain, error bars represents the standard error of the mean. (C) The diameter of *lgg-1::gfp *positive seam cells was measured from photographs using the ImageJ software as in Fig. 2. At least 40 cells were scored for each strain. Error bars represent the standard error of the mean.

We wished to test if the small body size among the feeding defective mutants is associated with autophagy. To visualize autophagy we used the extrachromosomal reporter *lgg-1::gfp *which was introduced into the mutants *eat-3*, *pha-2 *and *pha-3*. *lgg-1 *is homologous to the *S. cerevisiae *gene Apg8/Aut7p [27] and mammalian MAP-LC3 [28] and is useful as a marker for autophagy. The gene is expressed in many cells, including neurons, pharyngeal muscle cells, intestinal cells, gonad, vulva and hypodermal seam cells [24]. During conditions where autophagy is absent the expression of *lgg-1::gfp *is diffuse; when autophagy occurs preautophagosomal and autophagosomal structures are seen as GFP-positive punctate areas (Fig. 5 and [24]). We studied the hypodermal seam cells in L3 larvae, a stage before the most noticeable difference in body length is seen between wild-type and the feeding defective mutants. We found that feeding mutants have many more GFP-positive punctate areas than wild-type control worms, indicating increased autophagy activity in the mutants. (Fig. 5A–B).

We also used the *lgg-1::gfp *reporter as a tool to estimate the size of the seam cells and found that, in three studied mutants, the reduction in body length (Table 2 and Fig. 4) correlates well with the reduced size of the seam cells (Fig. 5C).

### Feeding defective mutants exhibit decreased levels of fat deposits

**Figure 6:**
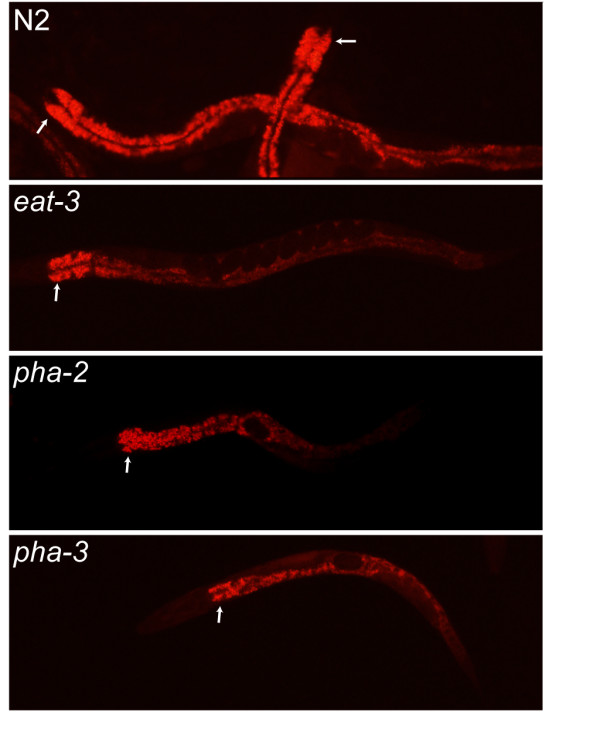
**Nile Red staining of fat stores in wild-type, *eat-3*, *pha-2 *and *pha-3 *adults**. L4 worms were grown on NGM plates containing the lipid-specific dye Nile Red for 24 hrs. The arrows point at the most anterior part of the intestine. (A) In wild type worms the fat granules in the intestine fluoresced brightly. In (B) *eat-3 *(C) *pha-2 *and (D) *pha-3 *mutants the fluorescence was markedly decreased, indicating reduced levels of fat deposits.

We then wished to investigate if the increased autophagy correlates with depletion of fat stores in the feeding defective mutants. Fat deposits can be labelled by uptake of the vital dye Nile Red, which can conveniently be included in the culture plates. Wild type, *dbl-1, eat-3*, *pha-2, pha-3*, *sma-1 *and *tax-6 *worms at different stages were grown 24 hrs on plates containing the dye. No difference in the level of Nile Red staining could be detected between wild type and *dbl-1*, *sma-1 *or *tax-6 *at any stages of life. In contrast, 1-day old adults of the three feeding-defective mutants tested (*eat-3*, *pha-2 *and *pha-3*) showed a clear reduction in Nile Red staining compared to age-matched controls (Fig. 6). Nile Red staining in these same mutants was indistinguishable from controls during the larval stages (data not shown). These results indicate that fat stores present during the larval stages are depleted as post-embryonic development proceeds in feeding-defective mutants.

## Discussion

We show here that a variety of unrelated mutations in genes that cause a wide range of feeding defects in *C. elegans *also cause a reduction in body length, and that the effects of these mutations are generally additive with body-shortening mutations that act via other pathways, namely the DBL-1/TGF-β pathway, the SMA-1/Spectrin pathway or the TAX-6/calcineurin pathway.

Reduced body length is therefore a second general property of the feeding defective mutants. The first reported general property is their increased longevity, which was attributed to the presumed benefits of caloric restriction on lifespan [14,18]. The nematode *C. elegans *is thus an organism where caloric restriction causes both a decrease in body size, and an increase in longevity. It has been known for decades that caloric restriction produces smaller mice and rats that also have increased lifespan, and the same correlations hold true within human populations [20]. Our study indicates that this is true also for nematodes.

All the feeding defective mutants in our study are significantly shorter than wild-type at the L4 and adult stages. The reduced body length also correlated well with the reduced size of seam cells in the studied mutants. However just after hatching and before feeding starts, the difference in length is only minor and for one of the shortest mutants, *eat-1*, that exhibits 53% of wild-type length as an adult, there is no difference at all in length at the L1 stage. These results indicate that the decrease in body length is mostly a post-development consequence of food deprivation.

Entry into the *dauer *state is environmentally triggered by a combination of starvation and high pheromone concentration, a measure of crowdedness [29,30]. It was recently shown that autophagy is enhanced in *C. elegans *during dauer formation where it might play a role in the remodelling of the animal as it becomes a dauer larva [24]. Our results show that autophagy is also induced at the L3 stage in feeding defective mutants, even the presence of abundant food and low worm density. This is an interesting observation because of the proposed role of autophagy in the increased longevity of insulin receptor mutants or of animals subjected to caloric restriction [31].

The insulin pathway, acting via the phosphatidylinositol kinase PI3K/TOR, plays an evolutionarily conserved role as an integrator of growth factor signalling and nutrient availability to regulate metabolism and body/organ/cell size [32,33], and longevity [34]. In *C. elegans*, the *daf-2 *pathway (*daf-2 *encodes a homolog of the insulin receptor) regulates entry into the long-lived dauer state, which involves a dramatic change from a sugar to a fat-based metabolism, as well as developmental arrest, activation of the autophagy process and other changes [24,35]. *daf-2 *acts via *daf-16 *(a FOXO forkhead transcription factor homolog) to regulate *let-363 *(the *C. elegans *PI3K/TOR homolog) which in turn activates autophagy, fat storage and other effector genes [36,37]. Published literature on the effects of feeding defects on body length, and the results that we present here show that these same processes (body size, longevity, autophagy) are coupled to nutrient availability in *C. elegans *even when these worms do not actually enter the dauer state: feeding-defective mutants have short body/cell size, increased autophagy and, known from previous publications [14], increased longevity.

It is the varied nature of the feeding-defective mutations that together support the idea that it is the feeding defect itself that causes reduced body length: the only phenotypic aspect these mutations have in common is their impact on feeding. For example, *eat-2 *encodes a nicotinic acetylcholine receptor subunit that regulates the rate of pharyngeal pumping. The gene is only expressed in the junction between the pharyngeal muscle cells pm4 and pm5 and the allele studied here *(ad465) *is considered as a null allele [38]. The *eat-2 *mutants exhibit 66% of wild-type body length and, given the function and expression profile of the encoded protein, it seems evident that the feeding defect is the cause of the decrease in body length in that mutant.

*pha-2 *mutants on the other hand, exhibit a severe pharyngeal morphological defect in which the isthmus that is normally a narrow passage for food, is shortened and thickened. In *pha-2 *worms, bacteria are trapped within the isthmus and are poorly transferred further to the intestine. The *pha-2 *gene encodes a homeodomain transcription factor and is expressed in pharyngeal cells (pm4, pm5, I4, epithelial cells) but also in many cells outside the pharynx (extrapharyngeal neurons, intestinal cells, rectal cells; [39]). The functions of the extrapharyngeal expression of *pha-2 *are at present unknown. We have recently showed that in the only existing *pha-2 *allele, *ad472*, the mutation is located upstream of the start codon and impairs the expression of *pha-2 *specifically in the pharynx [40]. Thus, here again, it is clearly the pharyngeal defect of the *pha-2 (ad472) *mutant that is responsible for the shorter length. The molecular identity of *eat-10 *and *pha-3 *is not yet known and *eat-1 *and *eat-3 *have only proposed identities so far (Table 1).

In summary, we have shown that food uptake efficiency acts as a pathway regulating body length, and that this pathway is likely separate from the DBL-1/TGFβ pathway. Given that a reduced rate of food uptake also causes increased longevity [14], we are tempted to propose that the effects of mutations that impair food uptake in some way result in a partial activation of the *dauer *program via a non-TGF-β pathway such that growth is slowed or arrested, even as development into a long-lived fertile adult stage proceeds.

## Conclusion

1. *C. elegans *feeding-defective mutants have previously been reported to have an extended lifespan. We show here that they are also smaller than wild-type.

2. Feeding defective mutations generally behave as a separate pathway regulating body length in *C. elegans: *their effects on body size are generally additive with mutations in other pathways that regulate body length.

3. *C. elegans *feeding-defective mutants have increased autophagy activity and decreased fat deposits.

## Methods

### Nematode strains and culturing

Maintenance and handling of worms were as described [41]. Wild-type parent strain used was the *C. elegans *Bristol variety strain, N2, [42]. All strains were cultured at 20°C. In addition to wild-type the following mutations were studied:

LG I: *rnt-1(ok351*).

LG II: *eat-2(ad465), eat-3(ad426)*.

LG III: *lon-1(e185), lon-1(wk50), sma-2(e502), sma-3(e491)*.

LG IV: *eat-1(ad427), egl-4(ad450), eat-10(ad606)*, *pha-3(ad607), tax-6(p675)*.

LG V: *dbl-1(nk3), sma-1(ru18)*.

LG X: *kin-29(oy38), pha-2(ad472)*.

### Generations of double mutants

Double mutants were generated by crossing males to hermaphrodites using standard techniques [41] and scoring progeny for the expected phenotypes. An exception was the *rnt-1(ok351) *mutation for which double mutants were confirmed by PCR using the following primers:

5'-rnt-1: 5'-CATCGTCGGCTCATAATAAAACTGC-3'

3'-rnt-1: 5'-CGAGGAGAAGATGGTCGTTTTAAC-3'

The PCR reaction produces a 2162 bp fragment for amplification of the wild-type allele and a 662 bp fragment for the deletion allele.

### Length measurements of eggs, L1, L4 larvae and adults

To measure the lengths and widths of adults, L4 larvae were picked to fresh plates and incubated at 20°C for 48 hrs. For study of L4 larvae worms with an obvious white crescent surrounding the visible prospective vulva were chosen. All L1 larvae were picked, mounted and photographed within 1 hour after hatching. Only eggs containing 3-fold stage embryos were studied. Eggs and worms were mounted on 2% agarose pads (in M9 buffer) and paralyzed with a drop of 100 mM levamisole and examined and photographed with a Zeiss axioplan compound microscope, using Nomarski optics and an attached AxioCam digital camera. All length measurements were performed with the free Java image processing program ImageJ [43]. Larvae and adults were measured from the nose to the tail tip (Fig 2). Eggs were measured by tracing their circumferences. The body widths were measured at the position of the vulva, from side to side.

### Autophagy study

Wild-type worms carrying the extrachromosomal array *lgg-1::gfp *[24] were kindly provided by Beth Levine. The array was introduced into *eat-3*, *pha-2 *and *pha-3 *mutants by crossing. Worms were studied at 1000 × magnification using a Zeiss axioplan compound microscope equipped with fluorescent optics. GFP positive punctate regions were counted from photographs of lateral hypodermal seam cells of worms at the L3 stage.

### Nile red staining

Worms were cultured 24 hrs on NGM plates containing 1 ng/ml Nile Red (5H-benzo [α] phenoxazine-5-one, 9-diethylamino) seeded with OP50. Fat content was monitored by fluorescence microscopy (rhodamine channel). All worms were photographed at a fixed exposure time.

## Authors' contributions

CM maintained the worm strains, generated the double mutants, and measured them. CM also performed the autophagy and Nile Red experiments, and helped in writing a draft of the manuscript. MP participated in the design of the study and in its coordination and helped to write the manuscript. All authors read and approved the final manuscript.
